# Contribution of the Brief Family Strength–Oriented Therapeutic Conversation Intervention to Early Childhood Sleep Health: A Quasi-Experimental Study

**DOI:** 10.1177/10748407241313463

**Published:** 2025-02-13

**Authors:** Erla Kolbrun Svavarsdottir, Kristin Bjorg Flygerning, Anna Olafia Sigurdardottir

**Affiliations:** 1 University of Iceland, Reykjavik, Iceland; 2 Landspitali-The National University Hospital of Iceland, Reykjavik, Iceland

**Keywords:** family nursing, educational support and behavioral intervention, strength-oriented therapeutic conversations, advanced family-pediatric nurses, pediatric sleep health

## Abstract

The family context is an important factor for sleep health in early childhood. About 40% of children between 0 and 3 years have problems regarding sleep that can affect their development. The aim of this study was to investigate the contribution of the brief family strength–oriented therapeutic conversation intervention to early-childhood sleep health. Data were collected in 2020–2021 from 57 primary caregivers of young children with moderate or severe sleep difficulties. The intervention was delivered through (a) a one 60-min face-to-face family educational and support session and (b) one to six 20- to 40-min telephone sessions based on the families’ needs. After the intervention, primary caregivers reported greater family support, more helpful beliefs about their infant’s/child sleep patterns, better family quality of life (QOL), better self-regulation regarding learning to fall and staying asleep among infants, and longer sleep periods at night in infants. These findings may prevent infants sleep difficulties from increasing and becoming more serious.

## Introduction

In early childhood, sleep health is established through sleep and wake adjustment and contributes to the health of children. Hence, the parents and families of infants and toddlers are vital for the development and management of healthy sleep in childhood (El-Sheikh & Kelly, 2017; Hall et al., 2023). Healthy sleep is essential for development, well-being, and general growth of children. Covington et al. (2021) defined good sleep health among children as acceptable sleep duration, regular wake and sleep times, and good quality of the sleep, which lack the parents’ perception of sleep problems in their infants or toddlers. Similarly, Meltzer, Williamson, and Mindell (2021), who developed the Buysse (2014) model of sleep health based on the socio-ecological framework, highlighted that pediatric sleep health is a multidimensional construct; that is, it encompasses five different domains, namely, (a) awareness or sleepiness (e.g., alertness when awake), (b) timing and placement of sleep, (c) sleep effectiveness and continuity or the ease of falling asleep, (d) sleep duration, and (e) satisfaction with or quality of sleep. Furthermore, Meltzer et al. concluded that behaviors related to sleep are important to consider in young children and pointed out that while some domains such as bedtime routine and consistent bedtime schedule are important for children, other aspects do not stay the same across child development such as sleep duration.

Paruthi et al. (2016) recommend that young children aged 4 to 12 months need to sleep for 12 to 16 hours every 24 hr, whereas children aged 1 to 2 years needed to sleep for 11 to 14 hr to promote optimal health. Nevertheless, Covington et al. (2021) emphasized that about 40% of infants and toddlers aged 0 to 3 years have sleep problems that can affect their development, physical health, socioemotional adjustment, and cognition. Low quality of sleep in childhood can also increase the danger of cardiometabolic diseases, negatively affect socioemotional development in children, and predict insufficient sleep during adolescence and adulthood (Corkum et al., 2019; Covington et al., 2021; Hanafin, 2018; Owens & Moore, 2017). Therefore, improving early-childhood sleep health may have an impact on health in adulthood.

## Background

A systematic review of 16 studies on family-level factors and their contribution to sleep health in early childhood (e.g., duration, timing, variability), sleep processes, and sleep difficulties (e.g., perceived sleep problems) showed that the family context contributed to sleep health in early childhood (Covington et al., 2021). In particular, family stress or strain were found to be correlated with sleep problems and fragmented sleep, whereas household routines were linked to a longer sleep duration. Furthermore, family-level factors and poor marital quality were directly linked to sleep problems in young children, whereas higher marital satisfaction correlated with fewer sleep problems and longer duration of sleep among young children. In addition, most interventions for improving sleep among children, focused on bedtime routines and parent interactions with the child, where boosting interactions and attachment at bedtime, was found to be related to improved sleep quality and duration. Family-level factors, such as implementing daily routines to minimize disorders, increasing support (e.g., for parents that are single), and advancing marital and coparenting relationships, were also shown to be beneficial in improving sleep health in childhood. Establishing appropriate sleep routines among young children was helpful and contributed to adequate sleep and essential health.

A population-based study of 2,014 Norwegian mothers and their toddlers reported that short sleep time period, awakening at nights, and sleep-onset difficulties were found to be related to higher odds of socioemotional problems (Hysing et al., 2016). Similarly, another study of 10,090 Australian infants/preschoolers and their families revealed that moderate to severe sleep problems had an impact on 17% of infants and 14% of preschoolers (Martin et al., 2007). Infant sleep difficulties were related to poor health in parents and had more impact on severe psychological distress.

In a previous study focusing on correlations between bedtime behaviors of 44 English families and their children’s sleep, about one third of the parents expressed their children had sleep problems (Cook et al., 2023). Majority of the sleep difficulties in children were rooted in behaviors, and the most common and successful interventions were behavioral based.

In an overview systematic review involving five reviews of 48 studies (majority randomized controlled trial [RCT]) that included 1,037 to 3,680 participants (*N* = 11,491), behavioral interventions were found to increase sleep in infants and parents (Drozd et al., 2022). The interventions mainly focused on (a) teaching children to fall asleep by themselves, (b) informing the parents regarding their young child’s sleep needs and the sleep cycle (sleep health literacy), and (c) implementing positive sleep habits (Drozd et al., 2022). In addition, the interventions focused on minimizing parents’ disruptions at sleep onset and encouraged infants to maintain and to fall asleep by themselves. Establishing helpful routines regarding bedtime were found to be critical to prevent and to manage sleep problems. Behavioral interventions were recommended to be adapted by the individual families because they could help infants to sleep longer at night and were expected to be helpful regarding infants waking up at nights.

A randomized controlled trial analyzed 235 families living in Canada who had 6- to 8-month-old infants that were dealing with problems regarding sleep. Of these families about half were assigned to the control and the intervention groups, respectively (Hall et al., 2015). Data were analyzed before and again one and a half month after the intervention. The intervention consisted of information offered in groups (2 hr) and support calls (four calls) offered in over 2 weeks’ time, with a focus on changing the families’ cognition and behaviors to support infants self-soothing. Overall, 97% and 98% of the infants were in the intervention and control groups, respectively, and they woke up two times per night on average. The intervention benefited the parents’ assessment of the young child’s sleep difficulties and the parents’ depression, sleep, tiredness, and sleep cognition, when evaluated in comparison with those in the control group.

Recently, Cato et al. (2024) studied Swedish parents who received an educational intervention regarding their infants’ sleep problems. These parents were assigned to a control (*n* = 42) or to an intervention (*n* = 46) group. This study revealed that being in the intervention group, being satisfied with your own sleep and being a mother, was found to be associated with higher self-efficacy.

Field (2017) reviewed 62 studies on interventions for young children with problems regarding sleep. In this review, both improvements as well as short time and small effects were reported. Field concluded that substantial methodological problems existed in the literature, that is, only using parents’ reports and mixed-age samples. Interestingly, Meltzer, Wainer, et al. (2021) conducted a 120 studies review (scoping) on interventions for sleep difficulties among young children and identified a clear gap in the literature, such as the necessity for more research in variety of different racial/ethnic groups worldwide as well as on families of children with physical and psychiatric illnesses/disorders. Studies on different levels and different types of interventions, including different methods of delivery, the family as a unit, and longitudinal research design, are also needed.

While specific pediatric sleep interventions have been developed and tested (Hall et al., 2015; Skuladottir et al., 2021), we know little about the helpfulness of a nurse-led intervention offered by an advanced nurse (e.g., a clinical nurse specialist or a nurse practitioner) to families at outpatient clinics in university hospitals, to increase healthy sleep patterns of young children. The “better sleep better well-being” intervention was tested among 35 Nordic parents of young children with sleep difficulties in the community (Skuladottir et al., 2022). Two sessions of support and educational intervention (face-to-face) and one telephone call session was provided, with a focus on child development, interactions (parent–child), beliefs, quality of life (QOL), temperaments, patterns of sleep, and problems regarding sleep. It took about 1 month to deliver the intervention. After the intervention, the parents experienced significantly higher support for their family, their physical functioning was better, and they reported their infant to both have longer periods of sleep over the nights and that their beliefs regarding their infants sleep patterns were improved.

The brief “Family Strength-Oriented Therapeutic Conversation” (FAM-SOTC) intervention was developed as a support and an educational intervention to assist parents in managing moderate-to-severe sleep problems in infants, to prevent them from further developing disorders, and to promote better sleep health. The brief FAM-SOTC intervention was based on the Calgary Family Models (both the assessment model [CFAM] and the intervention model [CFIM]; Shajani & Snell, 2023; Wright & Leahey, 2013) and the Illness Beliefs Model (Wright & Bell, 2021). In these models, families are defined as a unit of individuals (two or more) who are connected through strong emotional links to each other and feel that they belong to each other, have a deep interest in each other, and want to be a part of each other lives (Shajani & Snell, 2023). Family nursing interventions, defined as actions based on clinical judgment and knowledge, are used when nurses work with families, focusing on the change or to maintain the affective, behavioral, or the cognitive domains of family functioning. The brief FAM-SOTC intervention focuses on the normal development of young children, temperament, sleep patterns of infants, and interactions between infants/toddlers and their parents. An advanced nurse practitioner at an outpatient pediatric sleep clinic in the University Hospital is expected to offer evidence-based information regarding infants’ sleep to families and to support the families dealing with sleep problems among their infants. In addition, the nurses need to have an advanced family nursing education to be able to apply the intervention regarding infant’s sleep problems to the families.

The purpose of the study is to investigate the contribution of the brief FAM-SOTC intervention to early-childhood sleep health during the COVID-19 pandemic when data collection was challenged by minimal social contact. Based on the review of the literature and the CFAM/CFIM (Shajani & Snell, 2023; Wright & Bell, 2021), we hypothesized that the primary caregivers of infants and toddlers with sleep problems (moderate-severe) who received the brief FAM-SOTC intervention would report improved sleep pattern beliefs, higher perceived support for the family, and better QOL after the brief FAM-SOTC intervention than before. The brief FAM-SOTC intervention was delivered through (a) a one 60-min session (face-to-face) and (b) one to six telephone sessions based on the families’ needs (two to three phone sessions per family on average). In addition, the following research questions were asked to gain a further understanding of the infants’ sleep difficulties post-intervention compared with pre-intervention: (a) Was there a significant difference in the primary caregivers’ perception of how the infants fell asleep at night post-intervention, when compared with pre-intervention? (b) Was there a significant difference in the primary caregiver’s perception of what she or he did when the infants woke up at night post-intervention, when compared with pre-intervention? (c) Was there a significant difference in the primary caregiver’s perception regarding how the infants fell asleep when he or she took naps during the day post the intervention, as compared with pre-intervention?

## Method

Data were collected from October 2020 to May 2021. An advanced practice nurse (APN), who specialized in sleep health of young children, offered the intervention at the outpatient pediatric sleep clinic in University Hospital.

The primary caregivers, who were either the mothers or fathers of infants/toddlers, were concerned about their children’s sleep and consequently contacted the outpatient pediatric sleep health clinic for services and advice regarding their children’s moderate-to-severe sleep problems. Subsequently, a research assistant contacted the families and introduced them to the study. If the parents were interested in participating, they received a consent form from the research assistant. After signing the consent form prior to the face-to-face session of the brief FAM-SOTC intervention, the families received an e-mail with a code for Research Electronic Data Capture (REDCap) software to answer a set of questionnaires. The primary caregivers answered these questionnaires using the REDCap software before and after the intervention. The primary caregivers also received phone call sessions (one to six telephone sessions, depending on the parents’ needs). The face-to-face session at the outpatient pediatric sleep clinic lasted for approximately 60 min, whereas the phone call sessions (two to three sessions on average) lasted for approximately 20 to 40 min per session. The second and third telephone sessions were offered at 2 to 4 weeks after the first session (face-to-face). After receiving the brief FAM-SOTC intervention, the families answered again the questionnaires at time two.

The inclusion criteria were as follows: parents (a) who were capable of reading and writing Icelandic; (b) who were worried/concerned about their children’s sleep or experienced difficulty in handling their young children’s sleep at home; and (c) who felt that they needed specific support and information to manage their young children’s moderate-to-severe sleep problems and made appointments at the pediatric sleep health clinic with the APN. In our study, moderate-to-severe sleep problems were defined according to the following definitions presented by Hall et al. (2023): (a) insufficient sleep was defined as when the infants slept for less than 12 to 16 hr over a 24-hour period; (b) disrupted sleep was defined as when the infants woke up every night, about 3 to 4 times every night or more often); and (c) poorly timed sleep and/or unhelpful sleep habits were defined as when there was a lack of sleep routines or rhythms over the timed sleep patterns and bedtime routines both during the day and at night. The exclusion criteria were as follows: (a) the parents had been or were receiving consultations or interventions regarding pediatric sleep problems from another health care professional and (b) the caregivers were unable to read or write Icelandic.

During data collection, the parents of 147 children were invited to participate in an intervention study. Among them, 54 parents did not answer the questionnaires prior to the intervention (they forgot to answer) and were thus excluded from the study. A total of 93 primary caregivers answered the questionnaires at time one; however, 11 primary caregivers canceled their appointments before the intervention (due to being sick or having flu symptoms) and consequently did not receive the intervention. Furthermore, 82 primary caregivers received the brief FAM-SOTC intervention; however, 25 primary caregivers did not answer the questionnaires at time two (they indicated they did not have the time to answer). Ultimately, the final sample included 57 primary caregivers who participated at both time one and time two in the intervention study. The TREND and TIDieR guidelines were used to report the findings.

## The Brief FAM-SOTC Intervention

The brief FAM-SOTC intervention was originally developed at the University of Iceland, School of Health Sciences, Faculty of Nursing and Midwifery (Svavarsdottir et al., 2012) but was further elaborated in clinical practice at the University Hospital. The program was developed and modified to fit the families of infants/toddlers with sleep difficulties (moderate-to-severe) at the pediatric sleep health clinic. The brief FAM-SOTC intervention program was based on the CFAM/CFIM (Shajani & Snell, 2023) and the FAM-SOTC intervention (Svavarsdottir & Gisladottir, 2019). This program aimed to inform families about development among children, sleep, temperament, relationships (parent–children), how to motivate and empower about behavioral issues (i.e., behavioral components). In addition, this program educated the families about how to apply their knowledge (e.g., about infant sleep difficulties) to interactional situations (i.e., child and parent interactional component). Table 1 summarizes the content of the brief FAM-SOTC intervention.

**Table 1.** Content of the Face-to-Face and Phone Call Sessions of the Brief FAM-SOTC Intervention.

***The APN offering the brief-FAM SOTC intervention*:** *The face-to-face session:* In the first session, the pediatric APN met the family at the outpatient pediatric sleep clinic. She began by introducing the purpose, drawing the family tree, and creating a family support network system. She then encouraged the family to describe their experience of being the caregiver of an infant with sleep problems and asked therapeutic questions. In addition, she informed the parents about normal sleep patterns in infants and reviewed the infants’ sleep patterns, as indicated in the diary. The APA also asked parents about their infants’ sleep difficulties, habits, and the family’s daily life. The APN informed the parents about what could disturb an infant’s healthy sleep patterns and how to handle sleep difficulties. Simultaneously, the APN and the parents discussed the interventions that would be best for the family. Issues such as infant and parent intimacy and the timing of sleep and waking periods were also discussed. In addition, self-soothing capability, parents’ reactions during waking up at night, and nursing interventions based on the age of the infant and how they could be applied were discussed. The psychology of sleep, normal arousal, nutrition, and the importance of using family support networks are also discussed. The APN also drew forward family strengths when appropriately offered evidence-based information, supported facilitating beliefs, challenged hindering beliefs, and discussed the support network. Finally, the APN presented the main conclusions. *The first phone-call session:* In this session, the APN obtained feedback on the parents’ work from face-to-face sessions and how the family progressed. The APN then continued to apply the content of the brief FAM-SOTC intervention (Steps 3–8) to the conversation as appropriate. The APN also used parents’ motivation and empowerment end strengths to keep up with what they felt was working to manage the infant’s sleep difficulties. The APN evaluated the intervention and discussed whether modifications or changes were required to obtain the child’s self-soothing capabilities and sleep rhythms. *The second to sixth phone call sessions:* In the subsequent sessions of the brief FAM-SOTC intervention, the APN assessed progress from the first *face-to-face session* and evaluated and mirrored the infants’ and parents’ reactions. These sessions focused on family support systems, their strengths, and parents’ beliefs regarding their infants’ sleep patterns. In addition, the child’s self-soothing capability and parents’ skills regarding their infants’ regular sleep rhythms. The nurse encouraged parents to express how things were going and how they felt at the same time as the APN showed empathy and understanding. Furthermore, the APN focused on strengthening the families’ beliefs about the infants’ sleep patterns, changes that might have occurred in family life, and what they believed had been most helpful. The APN also continued to apply the content of the brief FAM-SOTC intervention (Steps 3–8) to the conversation as appropriate, focusing on family relations and strength and on how the parents felt they were handling their infant or their toddler’s sleep problem.

*Note.* APN = advanced practice nurse; FAM-SOTC = family strength–oriented therapeutic conversation.

**Figure 1.**
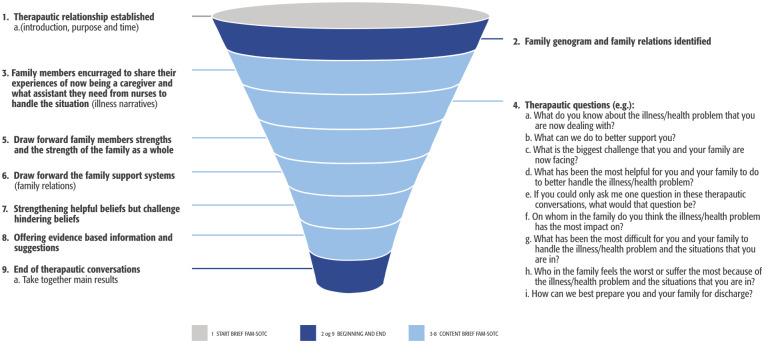
Nine-Step Brief Family Strength–Oriented Therapeutic Conversation (FAM-SOTC) Intervention.

The brief FAM-SOTC intervention consisted of nine steps (see Figure 1). The first session was a face-to-face session in which the APN introduced herself, described the aim of the brief FAM-SOTC intervention, and discussed the timeframe. Subsequently, the APN conducted a family tree and family relationships collaborating with the family. The core content of the brief FAM-SOTC intervention is described in Steps 3 to 8 in Figure 1, in which the steps were undertaken interchangeably as needed when offering the intervention. That is, the APN focused on encouraging the primary caregivers to share their story of being caregivers of young children with sleep problems, asked therapeutic questions, drew forward the family strengths with evidence-based information, strengthened the helpful infant sleep pattern beliefs of parents, challenged the constraining infant sleep pattern beliefs of parents, and worked with the family support network, as presented by the families. At the end of the brief FAM-SOTC intervention, the APN drew the core conclusion of the intervention.

During the two to three telephone interviews, the APN provided the primary caregivers with more support regarding how to manage their young children’s sleep problems (see Steps 3–8 in Figure 1). The APN ensured the fidelity of the study by describing in detail the intervention according to the manual, before each brief FAM-SOTC session, and offering the same or very similar content of the intervention to the families. However, at the same time, the FAM-SOTC intervention was tailored to the requirements of each family. Further details about the intervention can be requested from the first author.

## Measures

Data on the primary caregivers’ background and on the children’s demographics were gathered.

The Iceland Family Illness Beliefs Questionnaire is a valid seven-item scale that evaluates beliefs among members of the families who have a family member with health problems. The instrument evaluates sleep pattern beliefs (score range: 1–5) regarding the causes of sleep problems, effects, control, support, and suffering, with higher scores indicating greater assurance of family members’ beliefs. This instrument has satisfactory test–retest reliability and is valid (Svavarsdottir et al., 2020). Cronbach’s alpha for the questionnaire is .79 (Gisladottir & Svavarsdottir, 2016). In this study, Cronbach’s alpha was .68.

The Iceland Perceived Family Support Questionnaire is a 14-item instrument with two subscales, namely, emotional, and cognitive support. Scores range from low (1) to high (5). The measure is both reliable and valid (Svavarsdottir et al., 2020), with Cronbach’s alpha of .96 (total scale), .87 (cognitive subscale), and .94 (emotional subscale) (Sveinbjarnardottir et al., 2012). Cronbach’s alpha in this study was .93 for the total scale.

The 36-item PedsQL Family Impact Module was developed to measure pediatric health and its effect on QOL for the family. This measure has eight subscales which evaluate the families’ emotional/physical/cognitive/social functioning, worrying, communication, relationships, and daily activities. The measure is valid, with Cronbach’s alpha of .97 (Varni et al., 2004). In this study, Cronbach’s alpha was .94.

## Data Analysis

This study was a quasi-experiment with a pre–post one-group design. The data were normally distributed, as assessed using the Kolmogorov–Smirnov statistic (Field, 2024). However, the respondents’ rate (*n*) differed in the findings because of incomplete data. Only responses from each contributor were used to create the sum score for the main study variables. Descriptive analyses and paired *t*-tests were used to examine the data between the pre- and post-intervention scores. McNemar test was used to determine whether there was a change in dichotomous variables after the intervention regarding the infants/toddlers sleep habits (e.g., If the infant/toddler fell asleep at night, while fed with milk, through songs, when using white noise or when patted on the back or if he or she fell asleep by him or herself (rarely/never = 0, often/always = 1)). Data analysis was performed using IBM SPSS Statistics version 24.0 (IBM Corp., Armonk, NY, USA). Differences were considered statistically significant at *p* < .05 (Field, 2024).

## Ethical Considerations

This study was done in harmony with the moral values in the Declaration of Helsinki (World Medical Association, 2013) and was approved by the Ethics Committee of Landspitali National and University Hospital in Reykjavik Iceland in 2018 (VSN-18/2018). The data were stored in a secure location at the Social Science Research Institute of the University. Only authors had access to the data.

## Results

**Figure 2.**
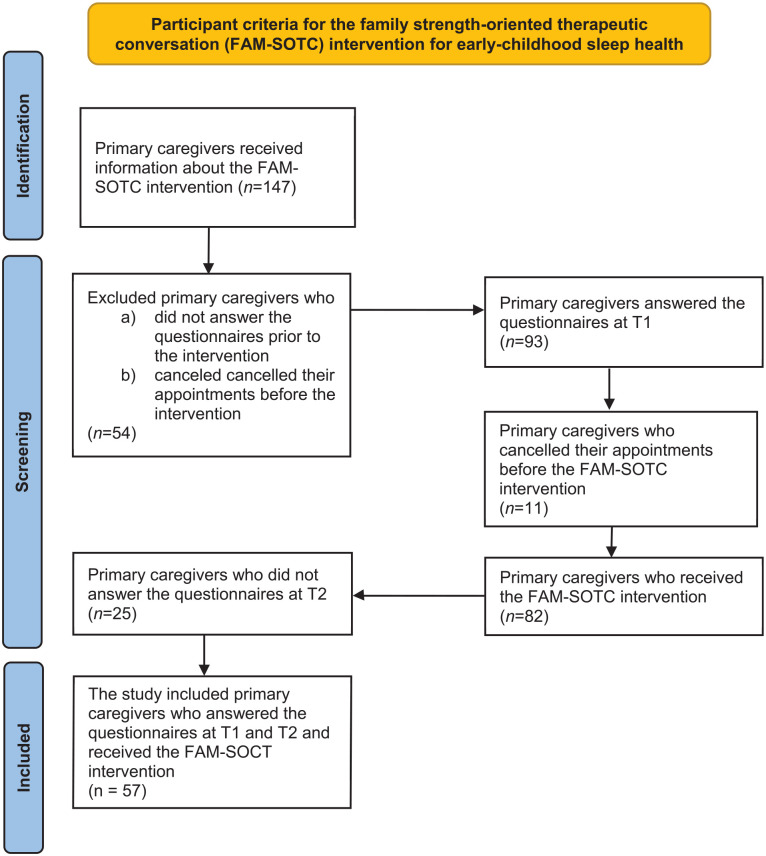
PRISMA Flow Diagram Regarding Participants. *Source.* Page et al. (2021).

There were 147 primary caregivers of infants with sleep problems that were introduced to the intervention research; however, 93 primary caregivers signed and participated at time one. Of those, 57 caregivers participated before and after the FAM-SOTC intervention, resulting in a participation rate of 61% (Figure 2). All primary caregivers were mothers (*n* = 52) or fathers (*n* = 5) of infants. The caregivers’ ages ranged from 18 to 50 years, with the majority varying between 18 and 40 years in age (*n* = 51, 89.5%). Most caregivers had a university degree (*n* = 42, 75%), were married or cohabiting (*n* = 54, 94.7%), perceived their general health as good or very good (*n* = 53, 93%), and had not experienced any trauma in their families within the last 12 months (*n* = 49, 86%). There were more primary caregivers of boys (*n* = 33, 57.9%) who participated in the study. Majority of the infants were 5 to 18 months of age (*n* = 50, 87.8%), used pacifiers (*n* = 41, 71.9%), and were either breastfed alone or both breastfed and bottle-fed (*n* = 29, 50.9%) (see Table 2).

**Table 2.** Demographics of Primary Caregivers (N = 57) of Young Children With Sleep Difficulties and Children’s Characteristics (n = 57).

Background variables	*n*	%
Primary caregivers (*N* = 57)		
Mothers	52	91.2
Fathers	5	8.8
Primary caregivers’ age		
18–30 years	25	43.9
31–40 years	26	45.6
41–50 years	6	10.5
Education		
Secondary	1	1.8
College	10	17.9
University	42	75.0
Other	3	5.3
Marital status		
Married/cohabiting	54	94.7
Single	3	5.3
General health		
Very good	22	38.6
Good	31	54.4
Fair	4	7.0
Did anyone in your family experience any trauma in the last 12 months?		
Yes	8	14.0
No	49	86.0
Children’s age		
2–4 months	3	5.3
5–8 months	27	47.4
9–12 months	14	24.6
13–18 months	9	15.8
19–24 months	2	3.5
2–3 years	2	3.5
Children’s gender		
Boys	33	57.9
Girls	24	42.1
Feeding		
Breastfeeding only	16	28.1
Bottle/glass feeding only	28	49.1
Breastfeeding and bottle feeding	13	22.8
Pacifier		
Yes	41	71.9
No	16	28.1

*Note. n* varies due to missing data.

Among primary caregivers, their beliefs regarding infants’ sleep patterns showed a significant difference after the brief FAM-SOTC intervention compared with before (T1 *M*: 22.60, T2 *M*: 26.60; *t* = −6.28, *p* = .000). Similarly, a significant difference was also found in the caregivers’ perceived family support after the brief FAM-SOTC intervention compared with before when considering the total scale (T1 *M*: 29.70, T2 *M*: 51.40; *t* = −9.80, *p* = .000), cognitive support subscale (T1 *M*: 13.40, T2 *M*: 19.80; *t* = −7.78, *p* = .000), and emotional support subscale (T1 *M*: 16.40, T2 *M*: 33.00; *t* = −10.08, *p* = .000) (Table 3). That is, primary caregivers expressed more confidence in understanding the cause of infants’ sleep problems and their impact on families; additionally, they were more knowledgeable about which coping strategies were helpful in adapting to their experience of sleep disorders. The primary caregivers also reported being more experienced in handling infants’ sleep problems and in receiving more support from the APN following the FAM-SOTC intervention.

**Table 3.** Perceptions Regarding Infants’ and Toddlers’ Sleep Pattern Beliefs, Family Support, and Family Quality of Life Before and After the FAM-SOTC Intervention Among Primary Caregivers (N = 56) of Young Children With Sleep Problems (Using Paired t-Tests).

Outcomes	Baseline				After the FAM-SOTC intervention				*t-*value	*p*-value
	*N*	*M*	*SD*	*Df*	*n*	*M*	*SD*	*df*		
Primary caregivers (*n* = 56)										
Sleep pattern beliefs	52	22.60	4.63	51	52	26.60	4.41	51	–6.280	.000
Perceived family support (total)	53	29.70	13.31	52	53	51.40	14.21	52	–9.802	.000
Cognitive support	53	13.40	5.94	52	53	19.80	4.22	52	–7.781	.000
Emotional support	52	16.40	8.84	51	52	33.00	10.27	51	–10.083	.000
Family quality of life (total)	56	54.90	14.81	55	56	60.80	16.82	55	–3.062	.003
Physical	56	37.60	17.92	55	56	50.07	18.93	55	–4.912	.000
Emotional	56	56.70	18.47	55	56	63.60	22.30	55	–2.295	.026
Social	56	50.00	25.25	55	56	54.02	24.90	55	–1.437	.156
Cognitive	56	48.90	21.38	55	56	53.70	24.28	55	–2.185	.033
Communication	56	64.90	21.77	55	56	67.90	22.55	55	– 1.067	.290
Worry	56	69.50	19.06	55	56	77.90	18.47	55	–3.024	.004
Daily activities	56	45.70	23.57	55	56	47.50	24.15	55	–0.719	.475
Family communication	56	68.00	18.65	55	56	68.80	22.50	55	–0.293	.771

*Note. n* varies due to missing data. FAM-SOTC = family strength–oriented therapeutic conversation.

The primary caregivers described that the family QOL (total score) was significantly higher after the brief FAM-SOTC intervention (T1 *M*: 54.90, T2 *M*: 60.80; *t* = −3.06, *p* = .003). In addition, the primary caregivers perceived their physical functioning (T1 *M*: 37.60, T2 *M*: 50.07; *t* = −4.91, *p* = .000), emotional functioning (T1 *M*: 56.70, T2 *M*: 63.60; *t* = −2.30, *p* = .026), and cognitive functioning (T1 *M*: 48.90, T2 *M*: 53.70; *t* = −2.19, *p* = .033) to be better post the brief FAM−SOTC intervention than before. The primary caregivers also expressed being less worried (T1 *M*: 69.50, T2 *M*: 77.90; *t* = −3.02, *p* = .004) after the brief FAM-SOTC intervention compared with before (Table 3).

**Figure 3.**
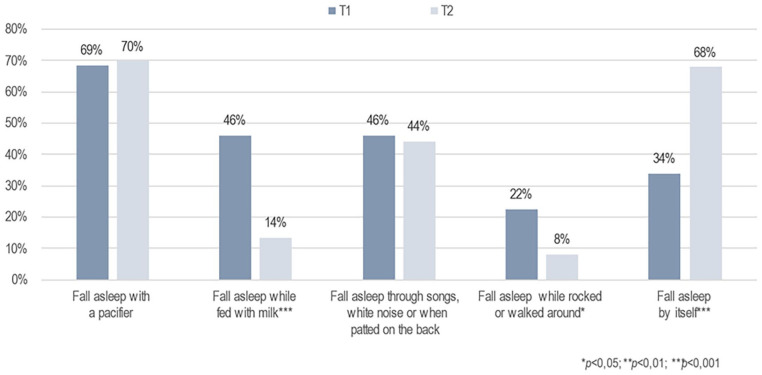
Perceptions of the Primary Caregivers (N = 56) Regarding How Infants and Toddlers Fell Asleep at Night.

**Figure 4.**
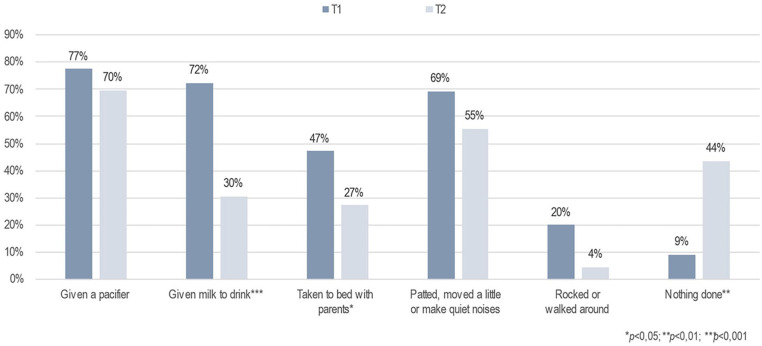
What Primary Caregivers (N = 56) Did When the Infants and Toddlers Woke Up at Night.

**Figure 5.**
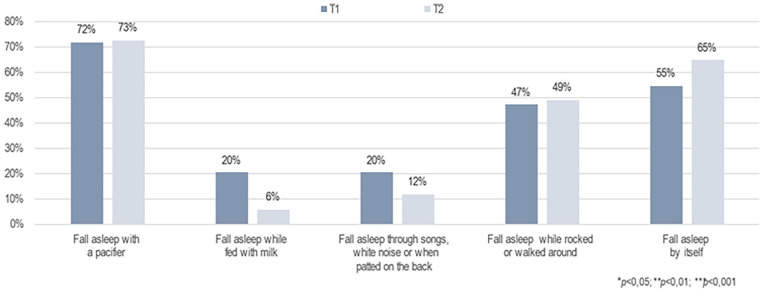
Perceptions of Primary Caregivers (N = 56) Regarding the Number of Infants’ Daytime Naps.

**Figure 6.**
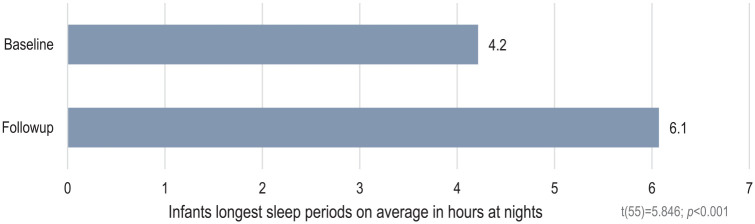
Average Longest Sleep Period in Hours at Night Among Infants and Toddlers.

Furthermore, the primary caregivers’ perception of infants’ sleep patterns at night changed significantly after the brief FAM-SOTC intervention compared with pre-intervention. After the intervention (at T2), the primary caregivers reported a significantly higher proportion of infants falling asleep while fed with milk or falling asleep on their own compared with before (T1) (*p* < .001) (Figure 3). The primary caregivers also reported fewer infants falling asleep while rocked or walked around (*p* < .05) after the intervention (T2) than before (T1) (Figure 3). In addition, the primary caregivers with infants who woke up at night reported significantly less given milk to drink, and less to take the infant to bed with them after the intervention (Figure 4). However, these caregivers reported a significantly higher frequency of doing nothing if their infants wake up at night (self-soothing) after the brief FAM-SOTC intervention compared with pre-intervention (Figure 4). With respect to daytime naps, the primary caregivers observed no significantly differences after the brief FAM-SOTC intervention compared with pre-intervention (Figure 5). Furthermore, the longest sleep period (in hours at night) of infants/toddlers on average increased significantly after the brief FAM-SOTC intervention compared with at baseline (before the brief FAM-SOTC intervention) (Figure 6).

## Discussion

Infants and toddlers depend on their primary caregivers to recognize their difficulties with sleep and seek support and evidence-based information from health care professionals to facilitate healthy sleep patterns. The present study hypothesized that the primary caregivers of infants and toddlers with sleep problems (moderate-to-severe) who received the brief FAM-SOTC intervention would report significantly improved sleep pattern beliefs, higher perceived family support, and better QOL following the intervention compared with before. The study findings are novel in that the primary caregivers considered themselves more confident in handling their young children with sleep difficulties (moderate-to-severe), reported being more confidence in understanding the cause of sleep problems and its impact on the family unit, and were more knowledgeable about helpful coping strategies to adapt to sleep disorders. In addition, the primary caregivers felt that their families were both cognitively and emotionally better supported regarding their infant/child’s sleep health by the APN after the intervention, compared with the traditional health care services, that they had received previously at the well-child clinic at their community health care center.

The primary caregivers perceived that their QOL (i.e., physical, emotional, and cognitive functioning) was better after the intervention and that they were less worried after the intervention than at baseline. This finding highlights the importance of APNs in the outpatient pediatric sleep health clinics, where infants, toddlers, and their families are treated for moderate-to-severe sleep problems. It is worth mentioning that the evidence-based brief FAM-SOTC intervention (Svavarsdottir & Gisladottir, 2019) was offered within an advanced clinical nurse practice. The brief FAM-SOTC intervention was not only beneficial to families of young children with sleep problems (moderate-to-severe) but was also effectively applicable to the outpatient pediatric sleep clinic. This finding is consistent with the call for nurses to promote healthy sleep in all populations, considering that healthy sleep is essential to the health and QOL of young children and adults (Hall et al., 2023). Hall et al. emphasized the importance of educating nurses after their BSc training in to prepare them in promoting sleep routines, screening for problems regarding sleep and screening for other health situations that can have an impact on sleep, as well as offering sleep interventions to stimulate good sleep patterns or plans among young children.

The brief FAM-SOTC intervention was developed and tested on families of infants/toddlers with sleep problems when the COVID-19 pandemic was at its peak. Overall, the face-to-face session and two to three phone call sessions benefited the families. In other words, the primary caregivers’ perception of infants’ sleep patterns at night improved after the brief FAM-SOTC intervention and they reported fewer infants falling asleep while being fed with milk or rocked. The parents of infants who woke up at night also reported less given the infant a milk to drink and taking less the infant into bed with them, after the intervention. Interestingly, however, the caregivers reported doing nothing more often if their infants woke up at night (i.e., more self-soothing) after the intervention. Therefore, the severity of the young children’s sleep difficulties decreased post-intervention compared with at baseline; that is, the infants had less insufficient sleep (over a 24-hr period), their sleep was less disrupted, and their sleep habits, including sleep routines and rhythms, improved after the intervention. This finding is important in that it clearly shows the value of the brief FAM-SOTC intervention for parents of infants with moderate-to-severe sleep problems and is in agreement with the results reported by Skuladottir et al. (2022), who showed that the better sleep better well-being intervention for caregivers of young children with sleep difficulties resulted in mothers reporting better support for the family, improved beliefs regarding sleep pattern, increased functioning (physical), and longer infant sleep at nights, after the intervention.

The FES-BSBW (Skuladottir et al., 2022) intervention was developed and tested at community health care centers for families who were worried about their infants’ sleep, whereas the brief FAM-SOTC intervention was offered at the outpatient pediatric sleep clinic for caregivers of young children with sleep difficulties. Nevertheless, both interventions were beneficial to the families and were based on the CFAM/CFIM (Shajani & Snell, 2023). In addition, both interventions focused on interactions (parent–child) and on the development of children, parents’ sleep patterns beliefs, temperament, and on the impact of child’s sleep difficulties on the parents’ QOL. These findings are promising and underscore the importance of the health care services offered by APNs in community and outpatient pediatric clinics, which can avoid sleep difficulties in early childhood from increasing into more serious health problems.

Our findings regarding the helpfulness of the brief FAM-SOTC intervention are also consistent with the results of Cato et al. (2024), who reported an educational intervention for families of young children with sleep difficulty, which involved offering education about sleep in infancy and the worth of attachment, as well as recommendation on sleep, feeding, and sharing bed, to benefit the parents regarding their own sleep and improve their self-efficacy. In addition, the findings from our current study are in harmony with findings by Covington et al. (2021), who found that family context contributed to sleep health in early childhood, and are in line with the results by Drozd et al. (2022), who showed in an umbrella review that interventions focusing on teaching children to fall asleep by themselves, informing caregivers about the need for sleep for children and educate them about the sleep cycle, as well as adapting positive sleep habits, increased sleep in both caregivers and the children.

The benefits of advanced nurse-led evidence-based interventions are encouraging and promising for nursing practice, where the content of the intervention and how it is delivered is of particular interest. This nine-step brief FAM-SOTC intervention can easily be administered to families of young children with sleep difficulties (moderate-to-severe), where the essence of the intervention is tailored to each family based on their needs. The core content of the intervention, that is, the third to eighth steps, can be used as needed and is interchangeable. The APN encourages parents to tell their story of being a caregiver of an infant/toddler with sleep problems, ask therapeutic questions, draw forward family strengths, offer evidence-based information, strengthen parents’ helpful infants’ sleep pattern beliefs, challenge parents constraining infants’ sleep pattern beliefs, and work with the family support network. In addition, in telephone sessions, the APN offers parents more support in handling their young children’s sleep problems. The brief FAM-SOTC intervention can be easily administered by an APN in a clinical setting and can, in that way, benefit the parents of infants/toddlers with sleep problems.

## Limitations

Parents who did not understand the Icelandic language were excluded, which could have limited the generalizability of the findings. The small sample size might have limited the generalizability of the results. This was a quasi-experimental study design, which also limited the generalizability. In addition, data were only analyzed from the primary caregiver’s perspective, not from both parents’ perspectives (no dyadic analysis); having a supportive partner can increase family well-being and QOL. So, the data were only collected at two time points (pre–post-intervention) with short follow-up measures, but not longitudinally. Longitudinal analysis could have provided more insight over time. Therefore, a brief FAM-SOTC intervention needs to be further tested with a bigger sample and in various family populations. Research with an experimental design or an RCT is needed to conclude about the effectiveness of the brief FAM-SOTC intervention.

## Conclusion

Creating a therapeutic alliance is crucial for APN practice at early-childhood sleep health clinics to help parents in handling sleep difficulties in their infants and toddlers. The contribution of the brief FAM-SOTC intervention was encouraging and valuable because of the benefit noticed after the brief intervention. Therefore, the brief FAM-SOTC intervention is worthy of evaluation in a clinical setting. The intervention added to the health of the family, given that the primary caregivers, who most often were the mothers, experienced higher support for their family, enhanced sleep pattern beliefs, higher family QOL, and better infants’ self-regulation following the brief FAM-SOTC intervention, which is critical for managing infant/toddler sleep problems. Overall, early-childhood sleep health should be prioritized in consultations because sleep is fundamental to children’s general health and the family well-being.

## References

[bibr1-10748407241313463] Buysse D. J. (2014). Sleep health: Can we define it? Does it matter? Sleep, 37(1), 9–17. 10.5665/sleep.3298 24470692 PMC3902880

[bibr2-10748407241313463] Cato K. Funkquist E.-L. Rosenblad A. K. (2024). Instrument development and an intervention to increase parents’ self-efficacy regarding their infant’s sleep. Sexual & Reproductive Healthcare, 39, Article 100944. 10.1016/j.srhc.2023.100944 38183709

[bibr3-10748407241313463] Cook G. Appleton J. V. Wiggs L. (2023). The relationship between parents’ cognitions, bedtime behaviours and sleep-related practices with their child's sleep. Journal of Sleep Research, 32(2), Article e13627. https://doi.org/doi.org/10.1111/jsr.13627 10.1111/jsr.13627 35567298

[bibr4-10748407241313463] Corkum P. Weiss S. Hall W. Brown C. Chambers C. Constantin E. Godbout R. Hanlon-Dearman A. Ipsiroglu O. Reid G. (2019). Assessment and treatment of pediatric behavioral sleep disorders in Canada. Sleep Medicine, 56, 29–37. 10.1016/j.sleep.2018.11.007 30555028

[bibr5-10748407241313463] Covington L. B. Patterson F. Hale L. E. Teti D. M. Cordova A. Mayberry S. Hauenstein E. J. (2021). The contributory role of the family context in early childhood sleep health: A systematic review. Sleep Health, 7(2), 254–265. 10.1016/j.sleh.2020.11.010 33436342

[bibr6-10748407241313463] Drozd F. Leksbø T. S. Størksen H. T. Wilhelmsen C. E. W. Slinning K. (2022). An overview of reviews for preventing and treating sleep problems in infants. Acta Paediatrica, 111(11), 2071–2076. 10.1111/apa.16475 35778903

[bibr7-10748407241313463] El-Sheikh M. Kelly R. J. (2017). Family functioning and children's sleep. Child Development Perspectives, 11(4), 264–269. 10.1111/cdep.12243 29731807 PMC5931738

[bibr8-10748407241313463] Field A. (2024). Discovering statistics using IBM SPSS statistics. SAGE.

[bibr9-10748407241313463] Field T. (2017). Infant sleep problems and interventions: A review. Infant Behavior & Development, 47, 40–53. 10.1016/j.infbeh.2017.02.002 28334578

[bibr10-10748407241313463] Gisladottir M. Svavarsdottir E. K. (2016). Development and psychometric testing of the Iceland-Family Illness Beliefs Questionnaire. Journal of Family Nursing, 22(3), 321–338. 10.1177/1074840716661593 27496811

[bibr11-10748407241313463] Hall W. A. Hutton E. Brant R. F. Collet J. P. Gregg K. Saunders R. Ipsiroglu O. Gafni A. Triolet K. Tse L. Bhagat R. Wooldridge J. (2015). A randomized controlled trial of an intervention for infants’ behavioral sleep problems. BMC Pediatrics, 15, Article 181. 10.1186/s12887-015-0492-7 PMC4643535 26567090

[bibr12-10748407241313463] Hall W. A. Keys E. Ou C. (2023). A call to action about nurses promoting healthy sleep. Sleep Medicine, 108, 53–54. 10.1016/j.sleep.2023.05.012 37327660

[bibr13-10748407241313463] Hanafin S. (2018). Sleep patterns and problems in infants and young children in Ireland. Child: Care, Health and Development, 44(3), 470–475. 10.1111/cch.12539 29230867

[bibr14-10748407241313463] Hysing M. Sivertsen B. Garthus-Niegel S. Eberhard-Gran M. (2016). Pediatric sleep problems and social-emotional problems: A population-based study. Infant Behavior and Development, 42, 111–118. 10.1016/j.infbeh.2015.12.005 26774862

[bibr15-10748407241313463] Martin J. Hiscock H. Hardy P. Davey B. Wake M. (2007). Adverse associations of infant and child sleep problems and parent health: An Australian population study. Pediatrics, 119(5), 947–955. 10.1542/peds.2006-2569 17473096

[bibr16-10748407241313463] Meltzer L. J. Wainer A. Engstrom E. Pepa L. Mindell J. A. (2021). Seeing the whole elephant: A scoping review of behavioral treatments for pediatric insomnia. Sleep Medicine Reviews, 56, Article 101410. 10.1016/j.smrv.2020.101410 33387973

[bibr17-10748407241313463] Meltzer L. J. Williamson A. A. Mindell J. A. (2021). Pediatric sleep health: It matters, and so does how we define it. Sleep Medicine Reviews, 57, Article 101425. 10.1016/j.smrv.2021.101425 PMC9067252 33601324

[bibr18-10748407241313463] Owens J. A. Moore M. (2017). Insomnia in infants and young children. Pediatric Annals, 46(9), e321–e326. 10.3928/19382359-20170816-02 28892546

[bibr19-10748407241313463] Page M. J. McKenzie J. E. Bossuyt P. M. Boutron I. Hoffmann T. C. Mulrow C. D. Shamseer L. Tetzlaff J. M. Akl E. A. Brennan S. E. Chou R. Glanville J. Grimshaw J. M. Hróbjartsson A. Lalu M. M. Li T. Loder E. W. Mayo-Wilson E. McDonald S. . . .Moher D. (2021). PRISMA 2020 statement: An updated guideline for reporting systematic reviews. BMJ, 372, Article n71. 10.1136/bmj.n71 PMC8005924 33782057

[bibr20-10748407241313463] Paruthi S. Brooks L. J. D'Ambrosio C. Hall W. A. Kotagal S. Lloyd R. M. Malow B. A. Maski K. Nichols C. Quan S. F. (2016). Recommended amount of sleep for pediatric populations: A consensus statement of the American Academy of Sleep Medicine. Journal of Clinical Sleep Medicine, 12(6), 785–786. 10.5664/jcsm.5866 27250809 PMC4877308

[bibr21-10748407241313463] Shajani Z. Snell D. (2023). Wright & Leahey’s nurses and families: A guide to family assessment and intervention (8th ed.). F. A. Davis.

[bibr22-10748407241313463] Skuladottir A. Sigurdardottir A. O. Svavarsdottir E. K. (2021). The better sleep better well-being programme: Educating and training community healthcare nurses in developing interventions for families of infants with moderate sleep problems—A pilot study. Scandinavian Journal of Caring Sciences, 35(1), 268–276. 10.1111/scs.12844 32240544

[bibr23-10748407241313463] Skuladottir A. Sigurdardottir A. O. Svavarsdottir E. K. (2022). The ‘Better sleep better well-being’intervention for parents of infants with moderate sleep problems: A quasi-experimental study. Nordic Journal of Nursing Research, 42(2), 85–92. 10.1177/20571585211044503

[bibr24-10748407241313463] Svavarsdottir E. K. Gisladottir M. (2019). How do family strengths-oriented therapeutic conversations (FAM-SOTC) advance psychiatric nursing practice? Journal of Nursing Scholarship, 51(2), 214–224. 10.1111/jnu.12450 30552746

[bibr25-10748407241313463] Svavarsdottir E. K. Kamban S. Konradsdottir E. Sigurdardottir A. O. (2020). The impact of family strengths oriented therapeutic conversations on parents of children with a new chronic illness diagnosis. Journal of Family Nursing, 26(3), 269–281. 10.1177/1074840720940674 32723122

[bibr26-10748407241313463] Svavarsdottir E. K. Tryggvadottir G. B. Sigurdardottir A. O. (2012). Knowledge translation in family nursing: Does a short-term therapeutic conversation intervention benefit families of children and adolescents in a hospital setting? Findings from the Landspitali University Hospital Family Nursing Implementation Project. Journal of Family Nursing, 18(3), 303–327. 10.1177/1074840712449202 22668768

[bibr27-10748407241313463] Sveinbjarnardottir E. K. Svavarsdottir E. K. Hrafnkelsson B. (2012). Psychometric development of the Iceland: Family Perceived Support Questionnaire (ICE-FPSQ). Journal of Family Nursing, 18(3), 328–352. 10.1177/1074840712449203 22821443

[bibr28-10748407241313463] Varni J. Sherman S. A. Burwinkle T. M. Dickinson P. E. Dixon P. (2004). The PedsQL™ family impact module: Preliminary reliability and validity. Health and Quality of Life Outcomes, 2(1), Article 55. 10.1186/1477-7525-2-55 PMC521692 15450120

[bibr29-10748407241313463] World Medical Association. (2013). Declaration of Helsinki: Ethical principles for medical research involving human subjects. Journal of the American Medical Association, 310(20), 2191–2194. 10.1001/jama.2013.281053 24141714

[bibr30-10748407241313463] Wright L. M. Bell J. (2021). Illness beliefs: The heart of healing in families and individuals. 4th Floor Press.

[bibr31-10748407241313463] Wright L. M. Leahey M. (2013). Nurses and families: A guide to family assessment and intervention (6th ed.). F.A. Davis.

